# Artificial intelligence-assisted three-dimensional preoperative planning with patient-specific cutting guides versus conventional planning and instrumentation in total knee arthroplasty: a prospective exploratory cohort study

**DOI:** 10.1186/s13018-026-07132-4

**Published:** 2026-08-14

**Authors:** Xueqi Liu, Hui Sun, Tianxiang Yang, Tao Ma, Yinbin Wang, Jun Li, Yi Wang, He Shang, Jian Wen, Desheng Chen

**Affiliations:** 1 Third Clinical Medical College of Ningxia Medical University, People’s Hospital of Ningxia Hui Autonomous Region, Yinchuan, 750004 People’s Republic of China; 2 Department of Orthopedics, People’s Hospital of Ningxia Hui Autonomous Region, Ningxia Medical University, Yinchuan, 750002 People’s Republic of China; 3 Department of Pain Management, The 2nd Affiliated Hospital, Jiangxi Medical College, Nanchang University, Nanchang, 330006 Jiangxi People’s Republic of China; 4 Department of Anesthesiology, People’s Hospital of Ningxia Hui Autonomous Region, Ningxia Medical University, Yinchuan, 750002 People’s Republic of China

**Keywords:** Total knee arthroplasty, Knee osteoarthritis, Gait analysis, Patient-specific instrumentation, Three-dimensional planning, Treatment outcome

## Abstract

**Background:**

Total knee arthroplasty (TKA) outcomes may be influenced by preoperative planning and accurate execution. This study compared an AI-assisted three-dimensional preoperative planning and patient-specific instrumentation (PSI) workflow with conventional planning and instrumentation for primary unilateral TKA. The AI-assisted workflow was a CT-based preoperative software platform for repeated virtual osteotomy, implant selection, component-position review, and virtual postoperative joint motion-state visualization; it was not robotic execution or a validated predictor of postoperative range of motion.

**Methods:**

In this single-center prospective exploratory cohort, 22 patients with knee osteoarthritis undergoing primary unilateral TKA were non-randomly allocated to an AI-assisted planning/PSI group (n = 11) or a Conventional group (n = 11). Outcomes were assessed preoperatively and at 2 and 6 months postoperatively. Clinical, perioperative, radiographic, and gait outcomes were compared using standard between-group and repeated-measures analyses. *P* values were interpreted as nominal because of the small sample size and multiple comparisons.

**Results:**

At 6 months, WOMAC scores were lower in the AI-assisted planning/PSI group than in the Conventional group [15.00 (14.00, 17.00) vs. 17.00 (15.00, 18.00); nominal *P* = 0.02]. KSS function scores were higher in the AI-assisted planning/PSI group at 2 and 6 months [80.00 (70.00, 90.00) vs. 55.00 (55.00, 60.00), and 80.00 (80.00, 90.00) vs. 70.00 (70.00, 70.00); both nominal *P* < 0.001]. Pain-related outcomes, HALP, drainage volume, and gait parameters showed directional between-group differences that were interpreted as exploratory and descriptive. Postoperative HKA deviation and HKA outlier rates were comparable, while coronal tibial component error was smaller in the AI-assisted planning/PSI group [0.4 (0.2, 0.7) vs. 0.9 (0.6, 1.1) degrees; nominal *P* = 0.018].

**Conclusions:**

In this exploratory cohort, AI-assisted planning with PSI was associated with selected short-term functional, perioperative, gait, and radiographic component-positioning differences, but not superior overall HKA alignment. Findings are preliminary and require confirmation in larger randomized or well-matched studies.

**Supplementary Information:**

The online version contains supplementary material available at https://doi.org/10.1186/s13018-026-07132-4.

## Introduction

Total knee arthroplasty (TKA) is a widely established surgical treatment for end-stage knee osteoarthritis that relieves pain, corrects deformities, and restores joint function [1, 2]. However, conventional TKA depends on accurate intraoperative execution of bone resection, alignment, and soft-tissue balancing, and variability in these steps may influence postoperative alignment and function [3, 4].

Digital technologies are increasingly used to support surgical planning and execution in orthopedics [4, 5]. In the present study, AI-assisted preoperative planning refers to a CT-based three-dimensional reconstruction, virtual osteotomy, implant planning, component-positioning review, and simulation workflow used before surgery. Its practical value lies in allowing the surgical team to repeatedly simulate osteotomy planes, review implant size and placement, and visualize the virtual postoperative joint motion state before entering the operating room. This process helps the team understand the planned surgical strategy in advance; however, it was not an autonomous robotic system, did not independently determine the final surgical plan, and did not provide true postoperative ROM data [6]. Patient-specific cutting guides were then designed to translate the finalized plan into intraoperative osteotomy execution [7, 8]. Previous studies suggest that patient-specific planning and instrumentation may support component positioning and lower-limb alignment, but their effects on operative time, blood loss, and functional recovery remain inconsistent [9, 10].

As an objective functional assessment tool, gait analysis quantifies postoperative recovery after TKA by measuring parameters such as stride length, walking speed, and joint kinematics [11]. Compared with traditional patient-reported and clinician-reported scores such as the Knee Society Score and the Western Ontario and McMaster Universities Osteoarthritis Index [12, 13], gait analysis provides an objective biomechanical description of postoperative movement patterns [14, 15]. However, the clinical importance of observed gait differences should be interpreted cautiously because such findings are exploratory and descriptive, and universally accepted minimal clinically important thresholds are not established for all gait metrics in TKA.

Perioperative physiological stress, including blood loss, nutritional depletion, inflammatory burden, and pain, may influence early recovery, especially in older patients [16]. For this reason, we explored the hemoglobin, albumin, lymphocyte, and platelet (HALP) score as a composite laboratory index reflecting perioperative nutritional and inflammatory status, although it has not been established as a standard outcome measure in TKA care [17–20].

Although studies of intelligent planning and patient-specific instrumentation in TKA are increasing, evidence integrating perioperative indicators, quantitative radiographic outcomes, patient-reported outcomes, clinician-reported scores, and objective gait analysis remains limited [21]. This prospective exploratory cohort study compared short-term clinical, radiographic, perioperative, patient-reported, and gait outcomes between patients undergoing TKA with AI-assisted three-dimensional preoperative planning plus patient-specific cutting guides and patients undergoing conventional planning and instrumentation at predefined postoperative follow-up time points. The purpose was to describe observed between-group differences and workflow feasibility, not to establish causal superiority.

## Materials and methods

### Study design and patient selection

This was a prospective exploratory cohort study conducted at a single tertiary orthopedic center. Patients who underwent primary TKA for end-stage knee osteoarthritis between March 2025 and May 2025 were consecutively enrolled at the Orthopaedic Center of the People’s Hospital of Ningxia Hui Autonomous Region. The study was approved by the institutional ethics committee (Approval No. 2025-GZR-007), and written informed consent was obtained from all participants. This manuscript was reported in accordance with the STROBE reporting principles for observational cohort studies, and a completed STROBE checklist is provided as Supplementary Table 1.

*Inclusion criteria were* (1) diagnosis of primary knee osteoarthritis based on clinical and radiographic findings; (2) indication for primary unilateral TKA; and (3) availability of complete perioperative data and follow-up records. *Exclusion criteria included* (1) inflammatory arthritis or post-traumatic arthritis; (2) history of prior knee surgery affecting alignment; (3) severe extra-articular deformities requiring corrective osteotomy; and (4) neuromuscular disorders or other conditions affecting gait.

Assignment to the AI-assisted planning/PSI workflow was not randomized. Before surgery, the operating surgeon explained the available surgical strategies to eligible patients and their family members. AI-assisted three-dimensional planning with patient-specific instrumentation was preferentially recommended for patients with more severe knee deformity, more complex bony anatomy, or anticipated technical difficulty, whereas younger patients with mild deformity and relatively neutral lower-limb alignment were generally not recommended for this workflow. Therefore, treatment allocation reflected surgeon-guided clinical selection rather than patient preference alone.

The AI-assisted planning/PSI workflow was supported by a regional assistance program for hospitals in western China and did not incur additional cost to the patients during the study period. Patient-specific cutting guides were produced using the hospital-based three-dimensional printing center, and no additional charge was applied for planning, printing, or sterilization. The workflow required approximately three additional days for preoperative CT acquisition, AI-assisted planning, guide fabrication, and sterilization before surgery. Patients were informed of this additional waiting time before admission. No patient initially allocated to the AI-assisted planning/PSI group crossed over to the Conventional group because of inability or unwillingness to wait for the additional workflow.

During the study period, 15 patients were counselled regarding the AI-assisted planning/PSI workflow. One patient did not undergo surgery because of perioperative complications, two patients did not choose the AI-assisted planning/PSI workflow, and one patient was excluded because of loss to follow-up. The remaining 11 patients were included in the AI-assisted planning/PSI group for analysis. Eleven patients treated with conventional planning and standard instrumentation during the same study period served as the Conventional comparison group.

### AI-assisted three-dimensional preoperative planning workflow

**Fig. 1:**
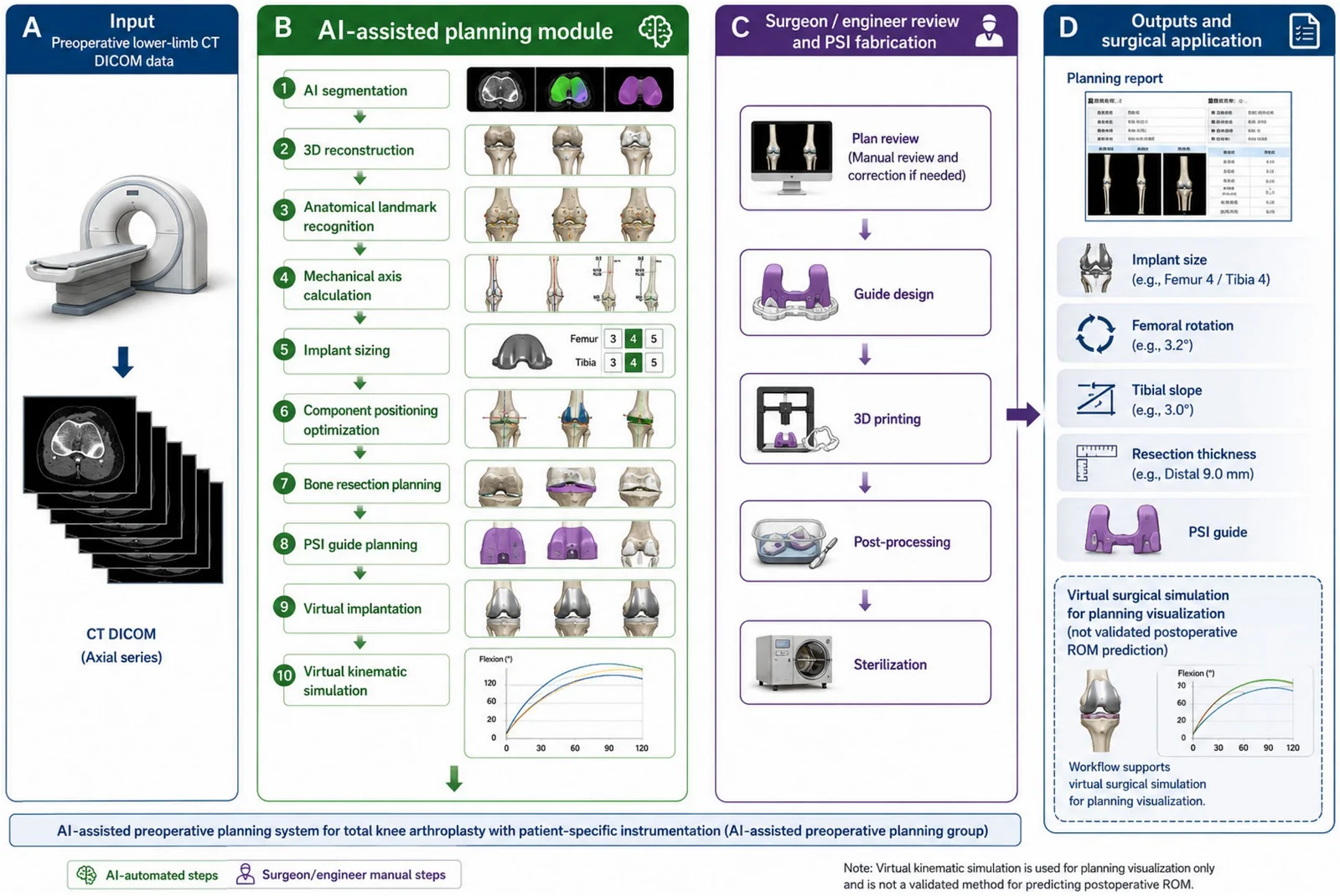
Overview of the AI-assisted preoperative planning and patient-specific instrumentation workflow for total knee arthroplasty. **A** Preoperative lower-limb CT DICOM data were imported as the input imaging data. **B** The AI-assisted planning module supported automated segmentation, three-dimensional reconstruction, anatomical landmark recognition, mechanical-axis calculation, implant-size selection, component-positioning review, bone-resection planning, patient-specific guide planning, virtual implantation, and virtual kinematic simulation. **C** The surgeon and engineering team reviewed and manually confirmed or adjusted the surgical plan, completed patient-specific guide design, and performed three-dimensional printing, post-processing, and sterilization. **D** The workflow outputs included the planning report, implant size, femoral rotation, tibial slope, resection thicknesses, patient-specific guide, and virtual surgical simulation for planning visualization. The virtual kinematic simulation was used only for intra-planning visualization and was not a validated method for predicting actual postoperative range of motion. *Abbreviations* CT, computed tomography; DICOM, Digital Imaging and Communications in Medicine; PSI, patient-specific instrumentation; ROM, range of motion

In the AI-assisted planning/PSI group, patients underwent hip-knee-ankle CT with thin-slice continuous acquisition (slice thickness 0.63 mm). CT DICOM data were imported into an AI-assisted arthroplasty planning platform for preoperative arthroplasty planning. The platform supported automated CT segmentation, three-dimensional reconstruction, anatomical landmark recognition, implant-size selection, bone resection planning, component-positioning review, and virtual implantation. During planning, the surgeon and engineering team could repeatedly review and adjust simulated osteotomy planes, implant selection, and component placement, and could review an animation-based virtual postoperative joint motion state before finalizing the plan (Fig. 1).

After the AI-assisted planning platform generated the preliminary segmentation, three-dimensional reconstruction, and virtual planning outputs, Mimics software (version 21.0, Materialise, Belgium) and 3-matic software (version 15.0, Materialise, Belgium) were routinely used during the model-checking and patient-specific guide-preparation stage. These tools were used to inspect the reconstructed bone models and segmentation boundaries, perform geometric clean-up or smoothing, prepare and check STL files, and support data transfer for subsequent patient-specific cutting-guide design. Their role was limited to auxiliary image/model processing and guide-preparation support, rather than serving as the core AI planning algorithm or determining the final surgical plan. The virtual motion output represented the postoperative joint activity state under simulated surgical conditions and was not actual postoperative ROM data or an independently validated prediction of actual postoperative ROM.

**Fig. 2:**
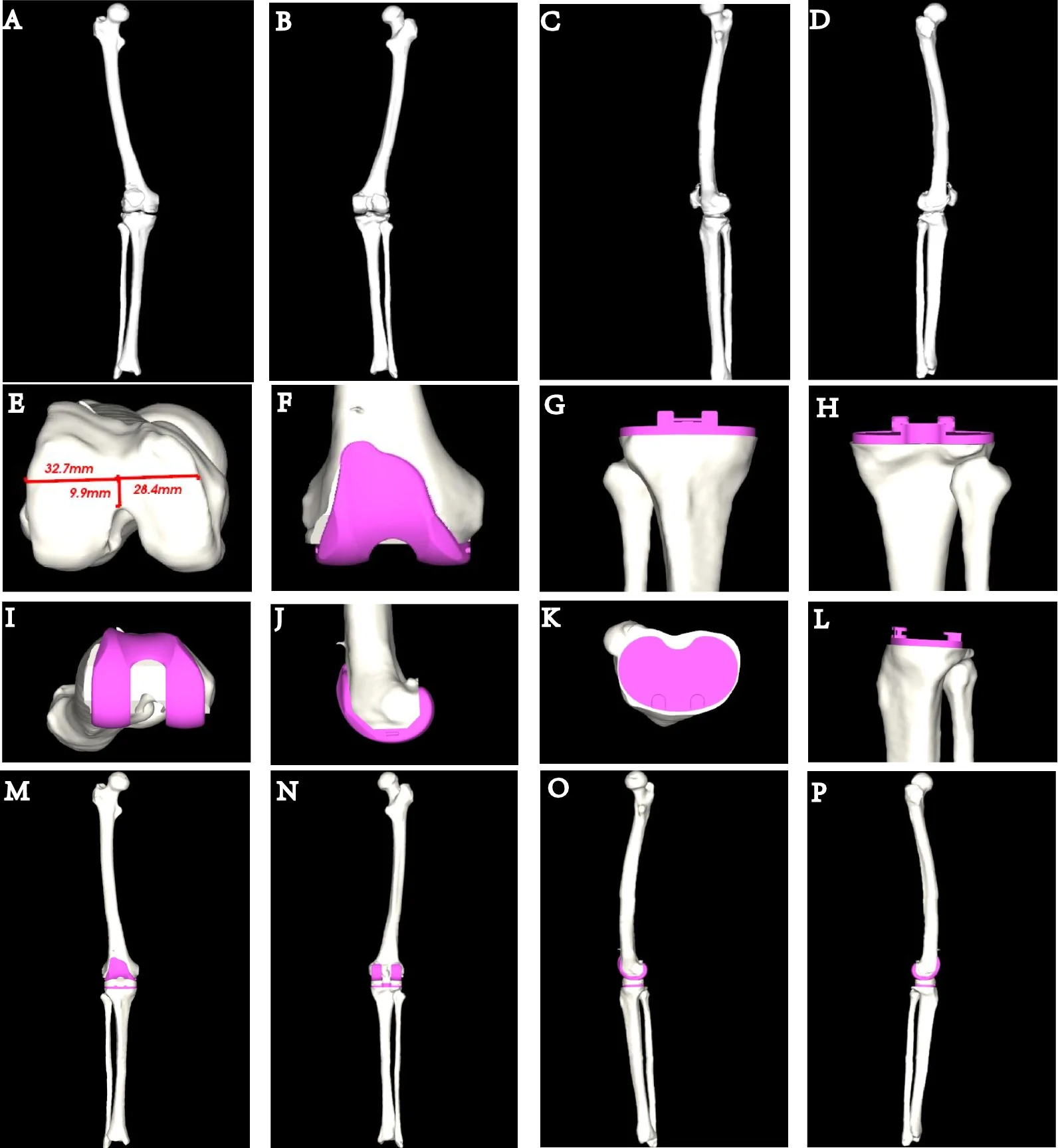
Representative three-dimensional preoperative planning outputs. **A–D** Acquisition of preoperative imaging data and three-dimensional reconstruction of the lower limb. **E–H** Identification of anatomical landmarks and measurement of key alignment parameters, including the hip-knee-ankle (HKA) angle. **I–L** Simulation of osteotomy planes, correction angle, and target mechanical axis based on preoperative planning. **M–P** Finalized surgical plan with virtual correction and virtual verification of the planned postoperative alignment. *Abbreviation* HKA, hip-knee-ankle

Virtual osteotomy planning was performed using predefined mechanical-alignment targets, including a 5 degrees valgus distal femoral cut and a proximal tibial cut perpendicular to the mechanical axis. Implant size, component position, and resection thickness were iteratively reviewed through virtual trialing, with the planning goal of supporting flexion–extension gap assessment, simulated alignment review, and implant surface coverage assessment. These outputs were used as intra-planning review parameters and did not constitute prospectively measured intraoperative accuracy, postoperative accuracy, or true clinical ROM endpoints. Representative planning outputs are shown in Fig. 2.

For patients assigned to the AI-assisted planning/PSI workflow, an additional preoperative workflow was required before surgery. After admission, lower-limb CT was obtained as an urgent preoperative examination, and the DICOM data were transferred to the planning workstation for software-based planning. AI-assisted segmentation, three-dimensional reconstruction, implant planning, and guide design were performed first, after which the hospital-based three-dimensional printing center fabricated and processed the patient-specific guide. The surgical plan and guide design were reviewed by the surgeon and engineering team before guide fabrication. Surgery was scheduled after guide post-processing and low-temperature plasma sterilization. In routine clinical practice during the study period, the overall turnaround time from urgent CT acquisition, data transfer, software planning, guide design, guide printing, post-processing, and sterilization to availability of the sterilized patient-specific guide was approximately three days. The main time-consuming steps were guide printing at the three-dimensional printing center, post-processing, and sterilization, rather than CT acquisition or software planning itself. Individual time stamps for CT acquisition, data transfer, AI segmentation, planning, guide design, printing, and sterilization were not prospectively recorded separately.

### Patient-specific cutting guide design

After validation of the virtual osteotomy planes, the cutting surfaces were exported as STL files and imported into Siemens NX software (version 2306, Siemens PLM Software, Germany). After mesh repair and conversion to STEP format, patient-specific cutting guides were designed with a standardized wall thickness of 2 mm, pinhole diameter of 2.0 mm, and edge chamfer of 0.5 mm.

**Fig. 3:**
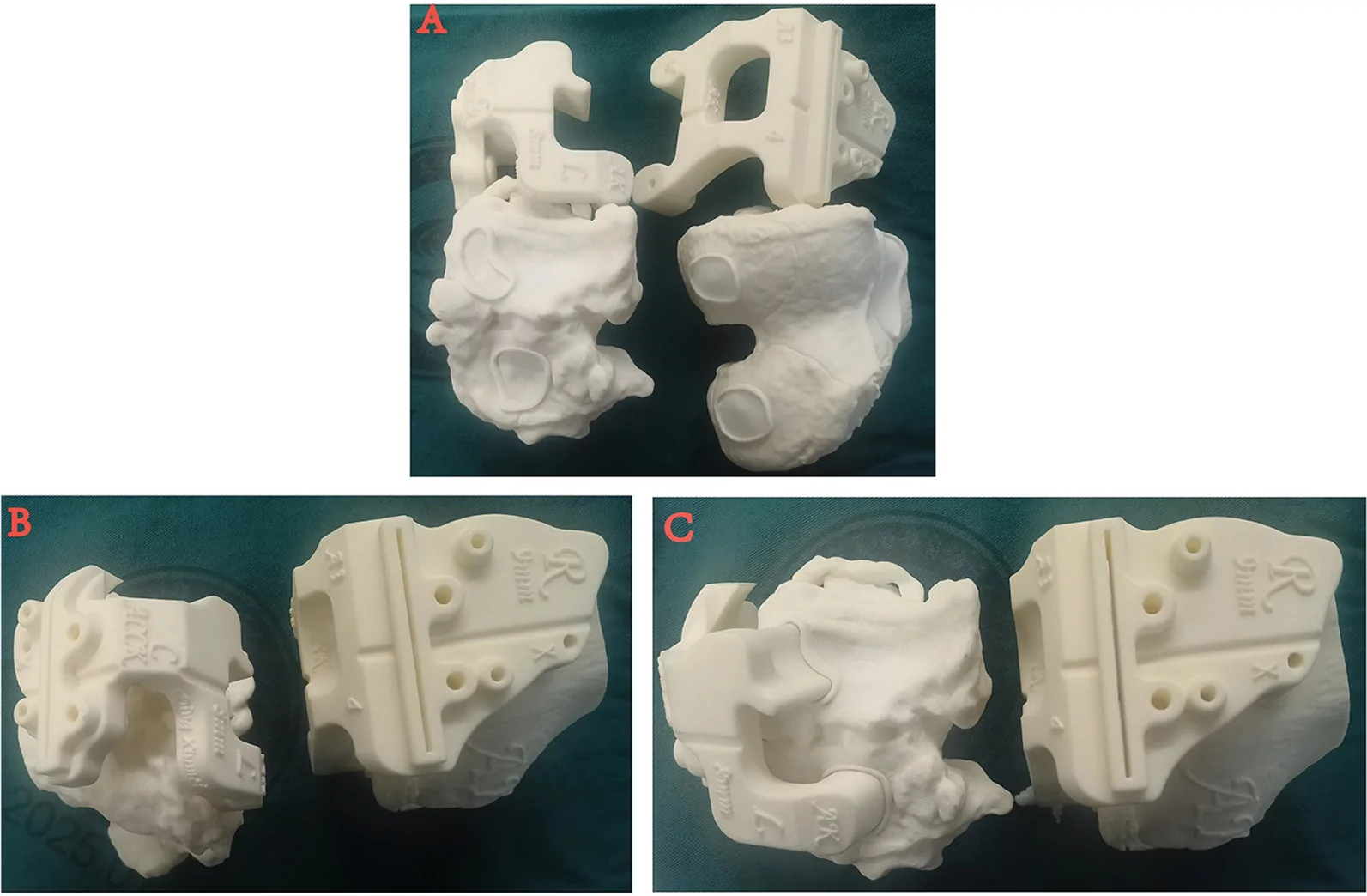
Design and structure of the patient-specific cutting guide. **A** Overall view of the patient-specific femoral and tibial cutting guides with the corresponding bone models. **B** Contact surface of the cutting guide, showing the individualized anatomical fitting region designed to match the bone surface. **C** Osteotomy slot and fixation-hole structure of the cutting guide, which was designed to guide saw-blade orientation and allow stable fixation during surgery

Design approval was based on acceptance criteria of registration error <  = 1 mm and maximum deviation <  = 0.5 mm in Geomagic Control X. The final three-dimensional printed parts were produced using selective laser sintering with medical-grade PA12 powder, followed by low-temperature plasma sterilization before surgery. The patient-specific cutting guide incorporated an individualized contact surface and integrated cutting slots to guide bone resection (Fig. 3).

### Surgical procedures

**Fig. 4:**
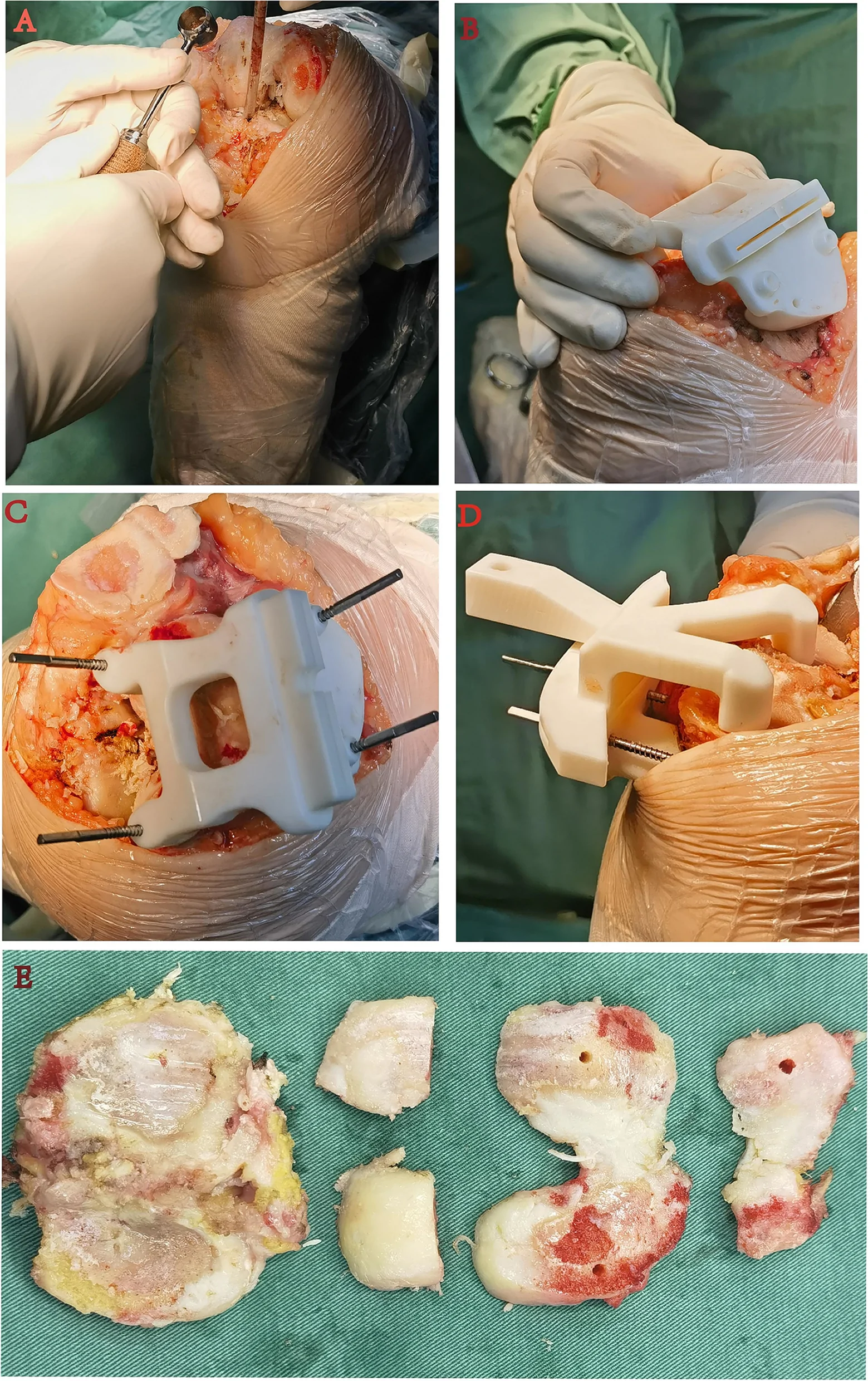
Intraoperative application of patient-specific cutting guides. **A, B** Placement of the patient-specific cutting guide on the bone surface according to the preoperative plan. **C, D** Fixation of the guide and osteotomy performed through the guide slot to facilitate execution of the planned bone resection. **E** Resected bone fragments after guide-assisted osteotomy

All procedures were performed by the same experienced surgical team to minimize inter-operator variability. In the AI-assisted planning/PSI group, TKA was performed using the finalized three-dimensional preoperative plan and patient-specific cutting guides. During surgery, the patient-specific guides generated from the preoperative plan were positioned using bony landmarks and fixed with Kirschner wires. Osteotomy was then performed through the guide slots to facilitate execution of the planned resections (Fig. 4). After guide-assisted bone resection and trial component placement, extension and flexion gaps were routinely assessed in the AI-assisted planning/PSI group as part of the standard intraoperative balancing procedure. When necessary, soft-tissue releases were performed stepwise to obtain stable and balanced medial–lateral gaps throughout extension and flexion, while avoiding excessive release or instability. Final implant positioning, polyethylene insert thickness, patellar tracking, and knee stability were confirmed intraoperatively by the operating surgeon. This workflow was intended to translate the preoperative plan into surgery and reduce repeated intraoperative adjustments; however, intraoperative accuracy and intramedullary canal violation were not quantitatively measured in this cohort.

In the Conventional group, TKA was performed using standard manual instrumentation without AI-assisted three-dimensional planning or patient-specific cutting guides. The same overall mechanical-alignment goal was used as in the AI-assisted planning/PSI group, aiming to restore a neutral lower-limb mechanical axis. For the femoral side, distal femoral resection was performed using an intramedullary alignment guide. The distal femoral valgus cutting angle was set according to preoperative radiographic assessment and the surgeon’s routine protocol, typically at 5 degrees to 7 degrees valgus relative to the anatomical axis of the femur. Femoral component rotation was determined using the posterior condylar axis as the primary reference, with approximately 3 degrees of external rotation, and was cross-checked with the transepicondylar axis, Whiteside’s line, and flexion-gap symmetry when applicable.

For the tibial side, proximal tibial resection was performed using a standard extramedullary alignment guide. The coronal tibial cut was targeted perpendicular to the mechanical axis of the tibia. The posterior tibial slope was set according to the implant design and the surgeon’s standard technique, generally aiming for approximately 3 degrees of posterior slope unless intraoperative anatomy or soft-tissue tension required adjustment. The conventional procedure followed a mechanical-alignment and measured-resection strategy, supplemented by intraoperative gap assessment and soft-tissue balancing. Extension and flexion gaps were assessed after bone resection and trial implantation. Soft-tissue releases were performed stepwise when necessary to obtain stable and balanced medial–lateral gaps throughout extension and flexion, while avoiding excessive release or instability. Final implant size, component position, polyethylene insert thickness, and soft-tissue releases were determined intraoperatively by the operating surgeon based on bone coverage, ligament balance, patellar tracking, and knee stability.

Both groups underwent TKA using the same implant system. Surgical approach, anesthesia protocol, perioperative management, and rehabilitation protocols were standardized across groups.

### Data collection and outcome measures

Perioperative, clinical, radiographic, functional, and gait-related data were collected preoperatively and during follow-up. Perioperative outcomes included operative time, postoperative drainage volume (0–24 h and 24–48 h), and the HALP score. Operative time was defined as intraoperative skin-incision-to-closure time and did not include preoperative CT acquisition, segmentation, planning, guide design, printing, or sterilization. HALP was explored as a composite laboratory marker of perioperative nutritional and inflammatory status rather than as a validated TKA-specific endpoint and was calculated as hemoglobin (g/L) x albumin (g/L) x lymphocyte count (/L)/platelet count (/L). Drainage volume was recorded at 24 and 48 h postoperatively.

Clinical functional outcomes were assessed using the KSS and WOMAC preoperatively and at 2 and 6 months postoperatively. The KSS includes a knee score and a function score, each ranging from 0 to 100, with higher scores indicating better status. The knee score assesses pain, stability, and range of motion, with deductions for flexion contracture, extension lag, and malalignment, whereas the function score reflects walking and stair-climbing ability [12]. WOMAC was collected using item-level 0–10 visual analog scale scoring (0 = none; 10 = extreme). For analysis and reporting, WOMAC total scores were linearly normalized to a 0–100 scale (normalized WOMAC = raw WOMAC/240 × 100), where higher scores indicate worse symptoms and greater functional limitation [13]. Pain was assessed using the numeric rating scale (NRS, 0–10). A severe pain episode was defined as NRS >  = 7 requiring rescue analgesia.

Postoperative active knee extension angle was recorded as a clinical knee extension parameter. The virtual postoperative joint motion state generated by the planning software was not analyzed as a clinical outcome because it was an intra-planning software simulation rather than true postoperative clinical ROM data. Actual postoperative flexion angle and total arc of motion were not systematically available; therefore, complete clinical ROM comparisons and comparisons between software-simulated motion state and actual postoperative clinical ROM were not performed. Postoperative active knee extension angle was considered a clinical recovery-related parameter rather than a mechanical limb-alignment measurement.

**Fig. 5:**
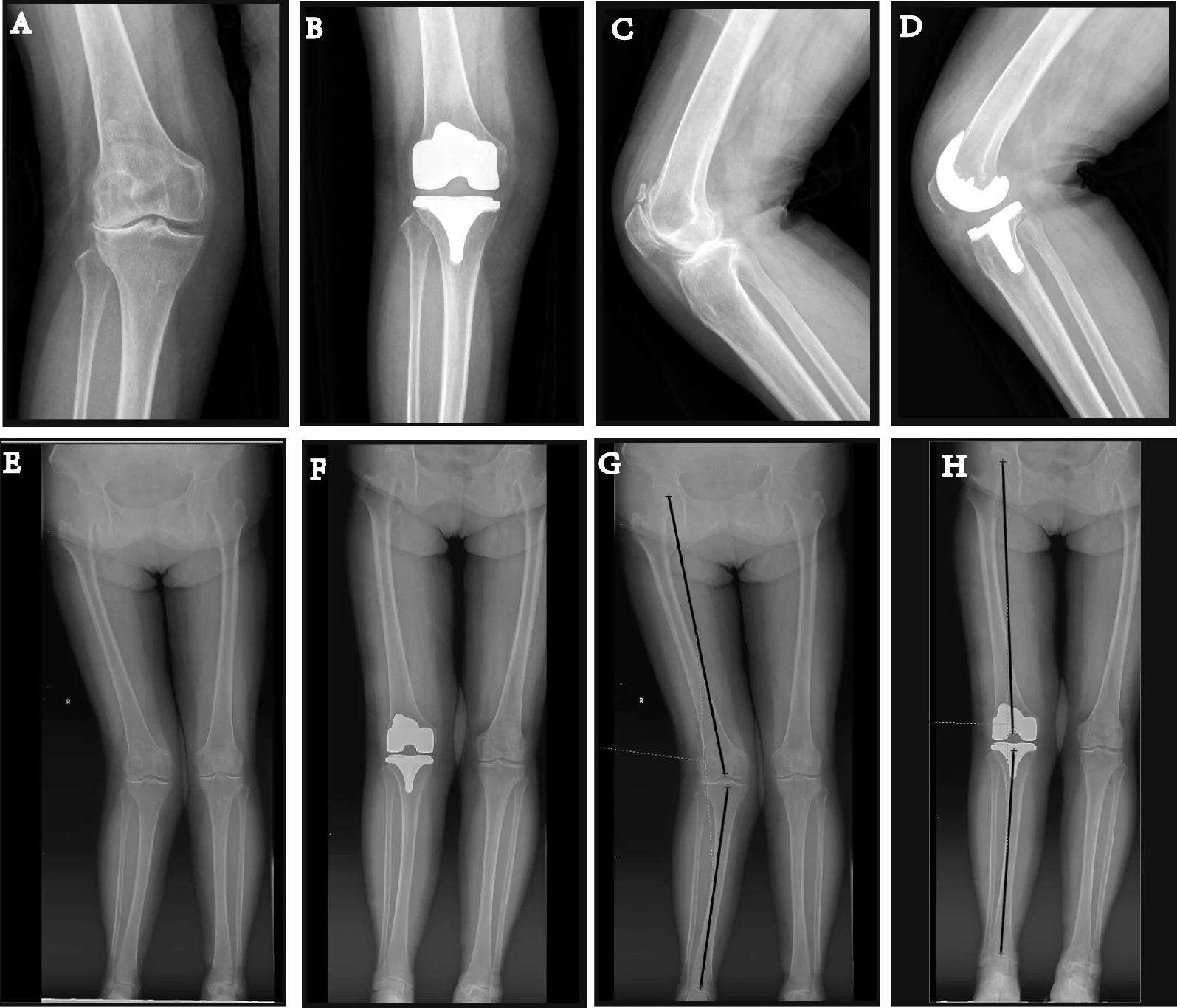
Representative preoperative and postoperative radiographs. **A–D** Knee radiographs in anteroposterior and lateral views before and after total knee arthroplasty. **E–H** Full-length standing radiographs before and after surgery, illustrating lower-limb alignment before and after total knee arthroplasty. Any overlaid lines are illustrative. Quantitative radiographic measurements were performed separately according to predefined outcomes

Quantitative radiographic outcomes were assessed to characterize achieved postoperative alignment and component positioning. Measurements included absolute postoperative HKA deviation from 180 degrees, coronal femoral component error, coronal tibial component error, posterior tibial slope error, medial proximal tibial angle (MPTA) absolute deviation from 90 degrees, and radiographic outlier rates beyond 3 degrees. These measurements characterized postoperative alignment and component positioning but did not validate the full accuracy of the preoperative software plan. Absolute preoperative HKA deviation from 180 degrees was used to quantify baseline coronal deformity severity regardless of deformity direction. Representative radiographs are shown in Fig. 5.

Outcome assessments were performed by research-team clinicians who were not involved in surgery and were blinded to group allocation. Patients and authorized proxies participated in counseling and consent; during follow-up, group assignment was not emphasized in an attempt to reduce expectation bias. Postoperative care pathways and follow-up schedules were standardized across groups.

### Gait analysis

Objective gait analysis was performed to complement functional assessments. Three-dimensional gait kinematics were acquired using a Qualisys optical motion-capture system and processed in Qualisys Track Manager (QTM). Marker trajectories were sampled at 100 Hz and filtered using a low-pass Butterworth filter with a 6 Hz cutoff frequency. A dynamic CAST protocol was used for lower-limb kinematic reconstruction. Pelvic markers were placed bilaterally on the anterior superior iliac spines and posterior superior iliac spines. During a static calibration trial, additional anatomical markers were placed on the medial and lateral femoral epicondyles and the medial and lateral malleoli to define knee and ankle joint axes. Foot markers were placed on the posterior calcaneus and the first, second, and fifth metatarsal heads. For dynamic walking trials, segment motion was tracked using rigid four-marker clusters attached to the posterolateral aspects of the thigh and shank to improve tracking robustness and reduce soft-tissue artifact; medial anatomical markers were used for static calibration only and were removed during dynamic trials to minimize occlusion and interference.

Patients walked barefoot along a level walkway at a self-selected comfortable speed. Each gait parameter was averaged over three successful trials. Gait outcomes included walking speed, cadence, step length, stride length, and the Gait Profile Score (GPS). Before analysis, gait data were checked for data-entry consistency and internal plausibility. Walking speed, cadence, step length, and stride length were exported from the same processed walking trials but were averaged according to the software’s event-detection and bilateral/single-limb conventions; therefore, arithmetic reconstruction of speed from group-level averaged cadence and step length may not be exact. Given the small sample size, gait analyses were interpreted as exploratory and descriptive directional postoperative trends rather than definitive evidence of gait efficacy or clinically meaningful gait superiority.

The GPS was calculated in Visual3D v6 Professional from the Movement Analysis Profile. The reference gait curves were derived from a laboratory-based healthy young adult cohort consisting of 23–25-year-old students with no history of lower-limb surgery, neuromuscular disease, symptomatic lower-limb disorder, or obvious lower-limb deformity. All reference participants and study patients were assessed using the same motion capture system, lower-body marker model, joint coordinate convention, and Visual3D processing pipeline.

GPS was calculated from standard lower-body kinematic variables included in the Movement Analysis Profile, including pelvic tilt, pelvic obliquity, pelvic rotation, hip flexion/extension, hip abduction/adduction, hip rotation, knee flexion/extension, ankle dorsiflexion/plantarflexion, and foot progression. GPS values were expressed in degrees, with lower values indicating less deviation from the laboratory-derived healthy young adult reference gait pattern. For unilateral TKA, the operated-limb GPS was used for the primary between-group comparison.

### Statistical methods

Statistical analyses were performed using SPSS software (version 25.0; IBM Corp., Armonk, NY, USA). Continuous variables were first assessed for normality using the Shapiro–Wilk test. Normally distributed data were presented as mean ± standard deviation and compared between groups using the independent-samples t-test. For normally distributed between-group comparisons, mean differences and approximate 95% confidence intervals were reported where they could be estimated from summary data. Non-normally distributed continuous variables were expressed as median (interquartile range) and analyzed using the Mann–Whitney *U* test; for these outcomes, group medians and absolute median differences were emphasized rather than *P* values alone. Categorical variables were compared using the chi-square test or Fisher’s exact test when expected counts were small. Confidence intervals were interpreted descriptively because of the exploratory design and limited sample size.

For quantitative radiographic error measurements, continuous variables were summarized as median [interquartile range] and compared using exact permutation Mann–Whitney tests because of the small sample size and non-normal distribution of error measurements. Hodges-Lehmann median differences with 95% confidence intervals and rank-biserial correlation coefficients were reported as effect estimates. Radiographic outlier rates were compared using Fisher’s exact test and reported with risk differences and 95% confidence intervals. An exploratory sensitivity analysis assessed absolute postoperative HKA deviation using an ordinary least-squares model adjusted for baseline absolute preoperative HKA deviation.

Repeated-measures analyses were used to evaluate group effects, time effects, and group-by-time interactions, with partial eta-squared reported as an effect-size measure. Because the study was exploratory and included a small sample with multiple outcomes and time points, *P* values were interpreted as nominal and descriptive rather than confirmatory. Effect estimates with 95% confidence intervals were reported where estimable, but no formal correction for multiple comparisons was applied. No multivariable adjustment, propensity-score matching, or weighting procedure was performed for the main clinical outcomes because of the limited sample size. No formal sample size calculation was performed.

## Results

### Patient flow, baseline characteristics, and operated side

A total of 22 patients were included in the final analysis: 11 in the Conventional group and 11 in the AI-assisted planning/PSI group. In the AI-assisted planning/PSI pathway, 15 patients were counselled regarding the workflow, one did not undergo surgery because of perioperative complications, two declined or did not choose the workflow, and one was lost to follow-up. No patient allocated to AI-assisted planning/PSI crossed over to conventional instrumentation because of the additional preoperative turnaround time.

Baseline demographic and clinical characteristics are summarized in Table 1. No strong statistical evidence of between-group differences was found in the measured baseline variables in this small sample. However, the preoperative KSS knee score showed a borderline between-group difference (*P* = 0.05), and the absence of statistical significance should not be interpreted as proof of baseline equivalence.

**Table 1:** Baseline data analysis results (n = 22)

Variable	Conventional (n = 11)	AI/PSI (n = 11)	Statistic	*P* value
Age (years)	69.18 ± 6.23	71.09 ± 6.43	*t* = − 0.71	0.490
Sex, female, n (%)	8 (72.73)	8 (72.73)	Fisher’s exact	1.000
Sex, male, n (%)	3 (27.27)	3 (27.27)	Fisher’s exact	1.000
BMI (kg/m^2^)	26.50 ± 3.49	28.35 ± 4.53	*t* = − 1.07	0.300
CRP (preoperative)	2.59 (2.16, 4.36)	4.00 (3.48, 5.12)	*z* = − 0.95	0.340
HALP (preoperative)	40.70 (24.21, 58.62)	61.16 (42.61, 72.24)	*z* = − 1.61	0.110
K–L stage (preoperative)	4.00 (3.00, 4.00)	4.00 (4.00, 4.00)	*z* = − 0.94	0.350
WOMAC (preoperative)	60.00 (56.00, 63.00)	63.00 (58.00, 67.00)	*z* = − 1.62	0.100
KSS score (preoperative)	51.00 (46.00, 52.00)	46.00 (45.00, 48.00)	*z* = − 1.92	0.050 †
KSS function score (preoperative)	65.00 (60.00, 70.00)	60.00 (60.00, 65.00)	*z* = − 1.46	0.140
HKA angle (preoperative, °)	171.00 (165.00, 174.00)	168.00 (163.00, 178.00)	*z* = − 0.63	0.530

Data are presented as mean ± SD, median (Q1, Q3), or n (%). Between-group comparisons used independent-samples *t* test for normally distributed variables or Mann–Whitney *U* test for non-normal variables. Categorical variables were compared using Fisher’s exact test when appropriate. HKA angle in this table is the raw preoperative angle and retains deformity direction. AI/PSI = AI-assisted planning with patient-specific instrumentation. †*P* = 0.05 indicates a threshold/borderline between-group difference and should be considered when interpreting postoperative comparisons

Operated side distribution did not differ statistically between groups (Fisher exact test, *P* = 0.198; Table 2).

**Table 2:** Distribution of operated side between groups (Fisher’s exact test)

Operated side	Conventional (n = 11)	AI/PSI (n = 11)	Total (N = 22)	*P* value
Right	8 (72.73)	4 (36.36)	12 (54.55)	0.198
Left	3 (27.27)	7 (63.64)	10 (45.45)	0.198

Values are presented as number (%). *P* values were calculated using Fisher’s exact test. Operated side distribution was reported separately because side dominance may influence gait assessment, although no statistical evidence of between-group difference was found in this cohort. AI/PSI = AI-assisted planning with patient-specific instrumentation

### Clinical and pain-related outcomes

WOMAC did not show statistical evidence of a between-group difference at 2 months (nominal *P* = 0.17). At 6 months postoperatively, the WOMAC score was lower in the AI-assisted planning/PSI group [15.00 (14.00, 17.00)] than in the Conventional group [17.00 (15.00, 18.00)] (absolute median difference, − 2 points; nominal *P* = 0.02), indicating a small observed between-group difference in this cohort (Table 3).

**Table 3:** Postoperative clinical and pain-related outcomes

Outcome	Conventional (n = 11)	AI/PSI (n = 11)	Statistic	*P* value
WOMAC score, 2 months postoperatively	26.00 (25.00, 27.00)	25.00 (22.00, 27.00)	*z* = − 1.36	0.170
WOMAC score, 6 months postoperatively	17.00 (15.00, 18.00)	15.00 (14.00, 17.00)	*z* = − 2.24	0.020*
KSS score, 2 months postoperatively	79.00 (76.00, 82.00)	82.00 (81.00, 83.00)	*z* = − 2.21	0.030*
KSS score, 6 months postoperatively	83.36 ± 1.91	85.45 ± 3.27	*t* = − 1.83	0.080
KSS function score, 2 months postoperatively	55.00 (55.00, 60.00)	80.00 (70.00, 90.00)	*z* = − 3.85	< 0.001**
KSS function score, 6 months postoperatively	70.00 (70.00, 70.00)	80.00 (80.00, 90.00)	*z* = − 3.34	< 0.001**
Time to first severe pain episode (h)	14.64 ± 5.87	23.91 ± 9.97	*t* = − 2.66	0.020*
Analgesic injections during hospitalization, n	4.00 ± 1.41	2.27 ± 1.95	*t* = 2.38	0.030*

Data are presented as mean ± SD or median (Q1, Q3). Between-group comparisons at each time point used independent-samples *t* test or Mann–Whitney *U* test, as appropriate. *P* values are nominal and exploratory because of the small sample size and multiple comparisons. Effect estimates and 95% confidence intervals for key comparisons are reported in the Results text where estimable. In repeated-measures analysis across time points, group-by-time interactions were significant for WOMAC (*P* = 0.0068, partial eta-squared = 0.221), KSS knee score (*P* = 0.00088, partial eta-squared = 0.297), and KSS function score (*P* < 0.001, partial eta-squared = 0.563). WOMAC is reported on a 0–100 normalized scale, where higher scores indicate worse symptoms. AI/PSI = AI-assisted planning with patient-specific instrumentation. *Nominal *P* < 0.05; **nominal *P* < 0.01

For the KSS knee score, the AI-assisted planning/PSI group had higher values at 2 months postoperatively [82.00 (81.00, 83.00)] compared with the Conventional group [79.00 (76.00, 82.00)] (absolute median difference, 3 points; nominal *P* = 0.03). At 6 months, the between-group comparison did not reach statistical significance (85.45 ± 3.27 vs. 83.36 ± 1.91; mean difference, 2.09 points; approximate 95% CI, − 0.30 to 4.48; *P* = 0.08).

The KSS function score was higher in the AI-assisted planning/PSI group at both 2 and 6 months postoperatively [80.00 (70.00, 90.00) and 80.00 (80.00, 90.00), respectively] than in the Conventional group [55.00 (55.00, 60.00) and 70.00 (70.00, 70.00), respectively] (absolute median differences, 25 and 10 points; both nominal *P* < 0.001).

Additional nominal between-group differences were observed in pain-related outcomes. The time elapsed until the first severe postoperative pain episode was longer in the AI-assisted planning/PSI group than in the Conventional group (23.91 ± 9.97 vs. 14.64 ± 5.87 h; mean difference, 9.27 h; approximate 95% CI, 1.88 to 16.66; *P* = 0.02). The number of rescue analgesic injections during hospitalization was lower in the AI-assisted planning/PSI group (2.27 ± 1.95 vs. 4.00 ± 1.41; mean difference, − 1.73 injections; approximate 95% CI, − 3.25 to − 0.21; *P* = 0.03). In repeated-measures analysis, the group-by-time interaction was significant for WOMAC (*P* = 0.0068, partial eta-squared = 0.221), KSS knee score (*P* = 0.00088, partial eta-squared = 0.297), and KSS function score (*P* < 0.001, partial eta-squared = 0.563).

### Inflammatory, nutritional, and early recovery-related outcomes

Postoperative inflammatory, nutritional, and early recovery-related outcomes are summarized in Table 4. Postoperative CRP did not differ statistically between groups (*P* = 0.31). In contrast, the postoperative HALP score was higher in the AI-assisted planning/PSI group than in the Conventional group (39.42 ± 11.04 vs. 26.80 ± 12.84; mean difference, 12.62; approximate 95% CI, 1.95 to 23.29; *P* = 0.02). This finding indicates an observed between-group difference in an exploratory laboratory index and may have been influenced by patient-related or unmeasured confounding factors.

**Table 4:** Postoperative inflammatory, nutritional, and early recovery-related outcomes

Outcome	Conventional (n = 11)	AI/PSI (n = 11)	Statistic	*P* value
CRP, postoperative	17.33 (10.25, 32.48)	11.71 (8.21, 22.21)	*z* = − 1.02	0.310
HALP, postoperative	26.80 ± 12.84	39.42 ± 11.04	*t* = − 2.47	0.020*
Operating time (min)	76.55 ± 15.81	76.09 ± 6.92	*t* = 0.09	0.930
Drainage volume, postoperative day 1	180.00 (150.00, 250.00)	150.00 (100.00, 160.00)	*z* = − 2.57	0.010*
Drainage volume, postoperative day 2	200.00 (100.00, 200.00)	100.00 (75.00, 150.00)	*z* = − 1.97	0.050†
Knee extension angle, postoperative (°)	173.36 ± 3.44	178.45 ± 3.24	*t* = − 3.57	< 0.001**
Contralateral knee extension angle, postoperative (°)	173.55 ± 5.65	173.36 ± 5.33	*t* = 0.08	0.940

Data are presented as mean ± SD or median (Q1, Q3). Between-group comparisons were performed using independent-samples *t* test or Mann–Whitney *U* test, as appropriate. *P* values are nominal and exploratory. Effect estimates and 95% confidence intervals for key normally distributed comparisons are reported in the Results text where estimable. Postoperative active knee extension angle was considered a clinical recovery-related parameter rather than a mechanical limb-alignment measurement. AI/PSI = AI-assisted planning with patient-specific instrumentation. *Nominal *P* < 0.05; **nominal *P* < 0.01. †*P* = 0.05 indicates a threshold/borderline between-group difference

Operative time did not differ statistically between groups (76.09 ± 6.92 vs. 76.55 ± 15.81 min; mean difference, − 0.46 min; approximate 95% CI, − 11.64 to 10.72; *P* = 0.93). The AI-assisted planning/PSI workflow required an additional preoperative turnaround time of approximately three days from CT acquisition to availability of the sterilized guide. This preoperative workflow time was not included in the operative-time measurement.

Postoperative drainage volume on the first day was lower in the AI-assisted planning/PSI group [150.00 (100.00, 160.00)] than in the Conventional group [180.00 (150.00, 250.00)] (absolute median difference, − 30 mL; nominal *P* = 0.01). Drainage volume on the second postoperative day was also numerically lower in the AI-assisted planning/PSI group [100.00 (75.00, 150.00)] compared with the Conventional group [200.00 (100.00, 200.00)] (absolute median difference, − 100 mL; borderline nominal *P* = 0.05).

Postoperative active knee extension angle was greater in the AI-assisted planning/PSI group than in the Conventional group (178.45 ± 3.24 degrees vs. 173.36 ± 3.44 degrees; mean difference, 5.09 degrees; approximate 95% CI, 2.12 to 8.06; *P* < 0.001). Contralateral knee extension angle did not differ statistically between groups (173.36 ± 5.33 degrees vs. 173.55 ± 5.65 degrees; mean difference, − 0.19 degrees; approximate 95% CI, − 5.08 to 4.70; *P* = 0.94). Postoperative knee extension angle was treated as a clinical recovery-related parameter rather than a quantitative radiographic alignment measurement.

### Quantitative radiographic outcomes

Quantitative radiographic assessment was performed to characterize baseline coronal deformity severity, achieved postoperative alignment, component positioning, and radiographic outlier rates (Tables 5 and 6). The AI-assisted planning/PSI group showed a tendency toward greater baseline coronal deformity, with a larger absolute preoperative HKA deviation from 180 degrees than the Conventional group [17.0 (10.5, 18.0) degrees vs. 9.0 (6.0, 13.0) degrees; median difference, 5.0 degrees; 95% CI, 0.0 to 9.0; exact *P* = 0.056]. Absolute correction of HKA deviation was larger in the AI-assisted planning/PSI group [11.0 (8.0, 13.5) degrees vs. 7.0 (5.0, 8.0) degrees; median difference, 5.0 degrees; 95% CI, 1.0 to 8.0; exact *P* = 0.010], which should be interpreted in the context of greater baseline deformity.

**Table 5:** Quantitative radiographic outcomes

Variable	Conventional (n = 11) Median (IQR), °	AI/PSI (n = 11) Median (IQR), °	Median difference (95% CI), °	Rank-biserial *r*	Exact *P* value
Absolute preoperative HKA deviation from 180°	9.0 (6.0, 13.0)	17.0 (10.5, 18.0)	5.0 (0.0 to 9.0)	0.48	0.056
Absolute postoperative HKA deviation from 180°	2.0 (1.0, 6.0)	3.0 (1.5, 5.0)	0.0 (− 4.0 to 3.0)	− 0.08	0.757
Absolute correction of HKA deviation	7.0 (5.0, 8.0)	11.0 (8.0, 13.5)	5.0 (1.0 to 8.0)	0.63	0.010
Coronal femoral component error (α)	0.7 (0.6, 1.1)	0.5 (0.2, 1.1)	− 0.2 (− 0.5 to 0.3)	− 0.18	0.488
Coronal tibial component error (β)	0.9 (0.6, 1.1)	0.4 (0.2, 0.7)	− 0.4 (− 0.8 to − 0.1)	− 0.59	0.018
Posterior tibial slope error	0.4 (0.2, 0.9)	0.3 (0.2, 0.5)	− 0.1 (− 0.5 to 0.1)	− 0.23	0.371
Absolute MPTA deviation from 90°	0.3 (0.2, 0.5)	0.2 (0.1, 0.3)	− 0.1 (− 0.3 to 0.1)	− 0.24	0.350

Values are presented as median [interquartile range]. Median differences were calculated as AI/PSI minus Conventional using Hodges-Lehmann estimation. *P* values were derived from exact permutation Mann–Whitney tests. Ninety-five percent confidence intervals for median differences were estimated using non-parametric bootstrap resampling. AI/PSI = AI-assisted planning with patient-specific instrumentation; HKA = hip-knee-ankle angle; MPTA = medial proximal tibial angle

**Table 6:** Radiographic outlier rates

Variable	Conventional (n = 11) n/N (%)	AI/PSI (n = 11) n/N (%)	Risk difference (95% CI), %	Odds ratio (95% CI)	Fisher’s exact *P* value
HKA outlier > 3°	5/11 (45.5%)	5/11 (45.5%)	0.0 (− 35.9 to 35.9)	1.00 (0.19 to 5.36)	1.000
Local component outlier > 3°	0/11 (0.0%)	0/11 (0.0%)	0.0 (− 25.9 to 25.9)	Not estimable	1.000
Any radiographic outlier > 3°	5/11 (45.5%)	5/11 (45.5%)	0.0 (− 35.9 to 35.9)	1.00 (0.19 to 5.36)	1.000
MPTA outlier > 3°	0/11 (0.0%)	0/11 (0.0%)	0.0 (− 25.9 to 25.9)	Not estimable	1.000

Values are presented as n/N (%). Risk differences were calculated as AI/PSI minus Conventional. Fisher exact tests were used for between-group comparisons. Odds ratios were not estimable when no events occurred in either group. AI/PSI = AI-assisted planning with patient-specific instrumentation; HKA = hip-knee-ankle angle; MPTA = medial proximal tibial angle

Postoperative absolute HKA deviation was comparable between groups [3.0 (1.5, 5.0) degrees vs. 2.0 (1.0, 6.0) degrees; median difference, 0.0 degrees; 95% CI, − 4.0 to 3.0; exact *P* = 0.757]. The HKA outlier rate > 3 degrees was identical between groups [5/11 (45.5%) vs. 5/11 (45.5%); risk difference, 0.0%; 95% CI, − 35.9% to 35.9%; Fisher exact *P* = 1.000].

For component positioning, the AI-assisted planning/PSI group demonstrated a smaller coronal tibial component error than the Conventional group [0.4 (0.2, 0.7) degrees vs. 0.9 (0.6, 1.1) degrees; median difference, − 0.4 degrees; 95% CI, − 0.8 to − 0.1; exact *P* = 0.018]. No statistically significant between-group differences were observed in coronal femoral component error, posterior tibial slope error, or MPTA absolute deviation from 90 degrees. No local component outliers > 3 degrees were observed in either group.

In an exploratory sensitivity analysis adjusted for baseline absolute preoperative HKA deviation, the adjusted group coefficient for postoperative HKA deviation was − 2.09 degrees in favor of the AI-assisted planning/PSI group, but this did not reach statistical significance (95% CI, − 4.63 to 0.44; *P* = 0.100). Baseline absolute preoperative HKA deviation was associated with postoperative HKA deviation (coefficient, 0.38 degrees per 1 degree; 95% CI, 0.15 to 0.62; *P* = 0.003; R-squared = 0.380). Representative radiographs are shown in Fig. 5.

### Gait analysis outcomes

At baseline, no statistical evidence of between-group differences was found for any gait parameter (all *P* > 0.05; Table 7). Internal checking did not identify data-entry discrepancies, but the gait data were interpreted cautiously because of the small sample size and software averaging conventions. Therefore, postoperative gait findings were treated as exploratory and descriptive between-group observations rather than definitive evidence of clinically meaningful gait benefit. At 2 months postoperatively, gait parameters showed nominal between-group differences. Walking speed was higher in the AI-assisted planning/PSI group than in the Conventional group (0.95 ± 0.12 vs. 0.78 ± 0.13 m/s; mean difference, 0.17 m/s; approximate 95% CI, 0.06 to 0.28; *P* = 0.003). Cadence was also higher (112.09 ± 12.11 vs. 98.55 ± 10.98 steps/min; mean difference, 13.54 steps/min; approximate 95% CI, 3.25 to 23.83; *P* = 0.012). Step length (0.58 ± 0.11 vs. 0.48 ± 0.10 m; mean difference, 0.10 m; approximate 95% CI, 0.01 to 0.19; *P* = 0.047) and stride length (1.15 ± 0.22 vs. 0.97 ± 0.20 m; mean difference, 0.18 m; approximate 95% CI, − 0.01 to 0.37; *P* = 0.047) were greater in the AI-assisted planning/PSI group. GPS was lower in the AI-assisted planning/PSI group (13.18 ± 2.92 vs. 18.41 ± 4.61; mean difference, − 5.23; approximate 95% CI, − 8.70 to − 1.76; *P* = 0.005), indicating less deviation from the reference gait pattern under the laboratory convention.

**Table 7:** Gait parameters between groups at different time points

Variable	Time	Conventional (n = 11)	AI/PSI (n = 11)	*t* statistic	*P* value
Walking speed (m/s)	Preoperative	0.69 ± 0.21	0.67 ± 0.12	0.273	0.787
	2 months postoperatively	0.78 ± 0.13	0.95 ± 0.12	− 3.329	0.003**
	6 months postoperatively	0.89 ± 0.17	1.09 ± 0.14	− 2.901	0.009**
Cadence (steps/min)	Preoperative	94.82 ± 14.87	92.27 ± 13.09	0.426	0.675
	2 months postoperatively	98.55 ± 10.98	112.09 ± 12.11	− 2.749	0.012*
	6 months postoperatively	105.18 ± 14.59	123.00 ± 8.85	− 3.462	0.002**
Step length (m)	Preoperative	0.52 ± 0.16	0.54 ± 0.14	− 0.311	0.759
	2 months postoperatively	0.48 ± 0.10	0.58 ± 0.11	− 2.118	0.047*
	6 months postoperatively	0.51 ± 0.13	0.63 ± 0.08	− 2.519	0.020*
Stride length (m)	Preoperative	1.04 ± 0.32	1.08 ± 0.28	− 0.311	0.759
	2 months postoperatively	0.97 ± 0.20	1.15 ± 0.22	− 2.118	0.047*
	6 months postoperatively	1.02 ± 0.26	1.25 ± 0.16	− 2.519	0.020*
Gait Profile Score (GPS)	Preoperative	25.13 ± 3.48	25.55 ± 4.24	− 0.253	0.803
	2 months postoperatively	18.41 ± 4.61	13.18 ± 2.92	3.181	0.005**
	6 months postoperatively	12.27 ± 2.58	9.05 ± 1.91	3.326	0.003**

Data are presented as mean ± SD. Between-group comparisons used independent-samples *t* tests at each time point. *P* values are nominal and exploratory. GPS values are in degrees, with lower values indicating less deviation from the reference gait pattern. Spatiotemporal variables were derived from the same processed walking trials and averaged using software event detection. AI/PSI = AI-assisted planning with patient-specific instrumentation; GPS = Gait Profile Score. *Nominal *P* < 0.05; **Nominal *P* < 0.01

**Fig. 6:**
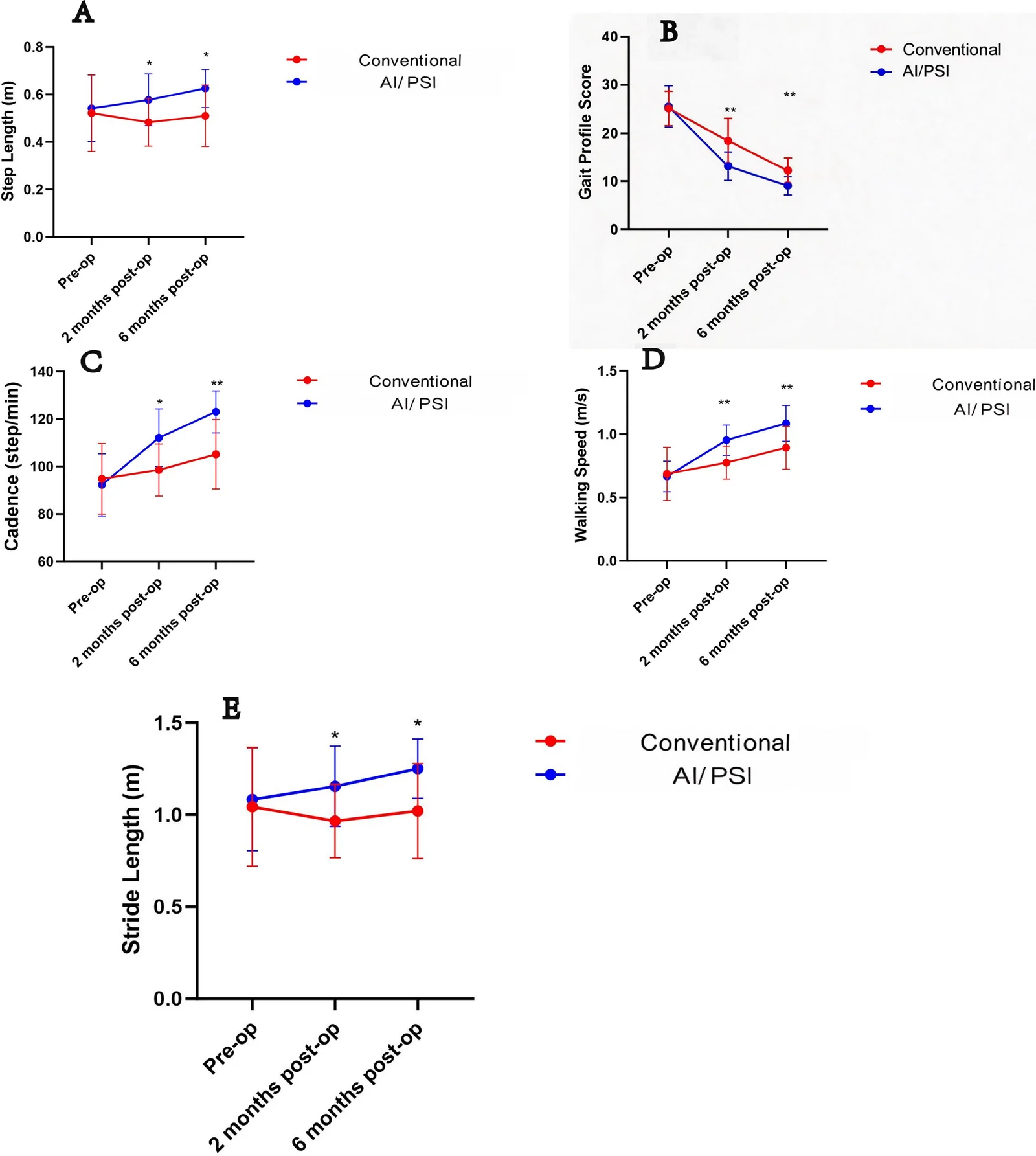
Changes in gait outcomes over time. Data are presented as mean ± standard deviation. Between-group comparisons at each time point were interpreted as nominal and descriptive because of the exploratory design and small sample size. **A** Step length over time. **B** Gait profile score (GPS) over time. **C** Cadence over time. **D** Walking speed over time. **E** Stride length over time. *Abbreviation* AI/PSI, AI-assisted planning with patient-specific instrumentation; GPS, gait profile score

At 6 months postoperatively, nominal between-group differences were also observed. Walking speed was higher in the AI-assisted planning/PSI group (1.09 ± 0.14 vs. 0.89 ± 0.17 m/s; mean difference, 0.20 m/s; approximate 95% CI, 0.06 to 0.34; *P* = 0.009), as was cadence (123.00 ± 8.85 vs. 105.18 ± 14.59 steps/min; mean difference, 17.82 steps/min; approximate 95% CI, 6.94 to 28.70; *P* = 0.002). Step length (0.63 ± 0.08 vs. 0.51 ± 0.13 m; mean difference, 0.12 m; approximate 95% CI, 0.02 to 0.22; *P* = 0.020) and stride length (1.25 ± 0.16 vs. 1.02 ± 0.26 m; mean difference, 0.23 m; approximate 95% CI, 0.04 to 0.42; *P* = 0.020) remained greater. GPS remained lower in the AI-assisted planning/PSI group (9.05 ± 1.91 vs. 12.27 ± 2.58; mean difference, − 3.22; approximate 95% CI, − 5.25 to − 1.19; *P* = 0.003). Figure 6 illustrates the trajectories of gait outcomes over time.

## Discussion

### Principal findings

This prospective exploratory cohort evaluated an AI-assisted preoperative planning and patient-specific instrumentation workflow for primary unilateral TKA. Similar technology-assisted TKA studies have increasingly focused on early functional recovery and clinical prediction, while broader orthopedic AI applications remain centered on decision support rather than autonomous surgical replacement [22, 23]. The main value of the workflow evaluated in the present study was not autonomous surgical execution, but the ability to support preoperative software-based simulation, repeated virtual osteotomy review, implant-size and implant-position review, guide fabrication, and virtual motion-state visualization before surgery.

Within this small non-randomized cohort, the AI-assisted planning/PSI group showed selected short-term differences in functional scores, perioperative indicators, component-positioning measures, and gait parameters compared with the Conventional group. These findings suggest that the workflow may be feasible as a planning-support strategy in selected patients, but they should be interpreted as exploratory clinical observations rather than evidence of causal superiority.

### Radiographic alignment and component positioning

Quantitative radiographic assessment was included to provide an objective description of postoperative alignment and component positioning. In this cohort, overall postoperative HKA deviation and HKA outlier rates were comparable between groups, whereas coronal tibial component error was smaller in the AI-assisted planning/PSI group. Therefore, the radiographic findings do not support a claim of superior global limb alignment, but they suggest a possible difference in tibial coronal component positioning.

This distinction is important because the AI-assisted planning/PSI group tended to have greater baseline coronal deformity. The larger absolute HKA correction observed in this group likely reflects greater baseline deformity rather than a standalone technical advantage. Importantly, all procedures in both groups were performed by the same senior surgical team. Under this condition, postoperative global alignment appeared comparable between the AI-assisted planning/PSI and Conventional groups, despite the AI-assisted planning/PSI group tending to include patients with more severe deformity. This suggests that preoperative simulation and patient-specific guide-assisted execution may provide a standardized planning and osteotomy reference during technically demanding TKA, potentially reducing exclusive reliance on intraoperative experience. However, this interpretation remains hypothesis-generating because the present study did not evaluate surgeons with different experience levels or a learning-curve effect.

After adjustment for baseline deformity, the trend toward lower postoperative HKA deviation did not reach statistical significance. These findings indicate that the relationship between preoperative simulation, guide-assisted execution, component positioning, surgeon experience, and postoperative recovery remains uncertain and should be tested in larger studies with predefined radiographic endpoints and surgeon-experience-related analyses. Implant-related stability and fixation considerations are also relevant when interpreting component-positioning outcomes after TKA [24, 25]. Previous studies have discussed the relevance of instrumentation approach, alignment strategy, component positioning, malalignment, and surgical factors in TKA biomechanics and functional outcomes [26–30].

### Functional recovery and gait outcomes

Between-group differences were observed in postoperative WOMAC and KSS-based outcomes. These findings are compatible with a possible role of standardized preoperative simulation, implant-position review, and guide-assisted execution in early functional recovery; however, because allocation was not randomized and residual confounding remains likely, the results should be interpreted as observed clinical differences rather than proof that the workflow caused superior function.

Objective gait analysis complemented patient-reported and clinician-reported outcomes by describing spatiotemporal gait parameters and GPS. Walking speed, cadence, step length, stride length, and GPS showed nominal between-group differences at postoperative time points. The data were internally checked and showed a consistent postoperative direction, but in this small cohort they should be interpreted as exploratory and descriptive gait observations rather than definitive evidence of clinically meaningful gait improvement. Postoperative gait after TKA may involve not only knee motion but also knee-related kinematics, cadence, stride characteristics, and compensatory movement patterns [31, 32]. The clinical importance of the observed gait differences remains uncertain because accepted minimal clinically important differences are not firmly established for several gait variables after TKA, particularly GPS.

### Postoperative drainage, pain, HALP, and early recovery

The AI-assisted planning/PSI group showed lower measured drainage volumes and a higher postoperative HALP score than the Conventional group. HALP is a composite index integrating hemoglobin, albumin, lymphocyte count, and platelet count, and has been discussed as a marker reflecting systemic nutritional-inflammatory status in clinical outcome studies [33]. A plausible perioperative explanation is that patient-specific guides may reduce reliance on intramedullary instrumentation, which has been discussed in relation to blood loss in TKA [26, 27]. In the present cohort, however, intraoperative blood loss, intramedullary canal violation, and transfusion-related endpoints were not directly quantified. Therefore, lower drainage volume should be interpreted as an observed perioperative difference rather than proof of reduced surgical trauma.

Pain-related outcomes also differed between groups, including a longer time to first severe pain episode and fewer rescue analgesic injections in the AI-assisted planning/PSI group. Postoperative pain control and enhanced recovery strategies are recognized contributors to early postoperative recovery [34, 35], but the present study cannot isolate the effect of planning/PSI from patient selection, expectation, rehabilitation engagement, and perioperative care factors. The higher postoperative HALP score may reflect differences in measured perioperative laboratory status; however, HALP is not a validated TKA-specific recovery endpoint and should be viewed only as an exploratory adjunct. Nutritional-inflammatory indices have been linked to outcomes in other surgical and medical settings [36, 37], but their role in arthroplasty recovery remains insufficiently established.

### Operative time, workflow feasibility, and clinical applicability

Operative time did not differ statistically between groups, but operative time captured only the intraoperative skin-incision-to-closure period and did not include the additional preoperative workflow required for AI-assisted planning/PSI. In the present institutional setting, this workflow consisted of urgent lower-limb CT after admission, data transfer to the planning system, software-based planning and guide design, three-dimensional printing-center fabrication and post-processing, low-temperature plasma sterilization, and surgical scheduling after the guide became available. The overall turnaround time from urgent CT acquisition, data transfer, software planning, guide design, guide printing, post-processing, and sterilization to availability of the sterilized guide was approximately three days. The main time-consuming steps were guide printing at the three-dimensional printing center, post-processing, and sterilization rather than CT acquisition or software planning itself. The study did not prospectively record the time required for each workflow step and did not compare the AI-assisted software-planning process with a fully manual modeling or planning workflow; therefore, whether AI-assisted planning shortened the software-planning process could not be evaluated. The broader clinical applicability of this workflow depends on access to thin-slice CT, AI planning software, engineering support, three-dimensional printing, sterilization capacity, and sufficient lead time before surgery. These requirements may limit implementation in smaller or resource-limited centers. Additive manufacturing and AI may support individualized surgical preparation, but implementation depends heavily on local infrastructure and workflow design [38, 39]. Patient counseling and shared decision-making remain important because the workflow introduces additional imaging, planning, and logistical steps [40]. The present study supports feasibility within the authors’ institutional setting but does not establish cost-effectiveness, workflow efficiency, or superiority across healthcare settings.

### Limitations and future directions

This study has several limitations. First, treatment allocation was not randomized. The AI-assisted planning/PSI workflow was preferentially recommended for patients with more severe deformity or anticipated surgical complexity, whereas patients with mild deformity and relatively neutral alignment were generally treated using conventional planning and instrumentation. This surgeon-guided allocation may have introduced selection bias and confounding by indication. Second, the sample size was small, which limited statistical power, precision, subgroup analysis, multivariable adjustment, and propensity-score matching. Third, multiple outcomes and time points were tested without formal correction for multiple comparisons; therefore, *P* values should be interpreted as nominal and exploratory. Although confidence intervals were reported where estimable, their precision remains limited by the small sample size.

Fourth, quantitative radiographic measurements characterized postoperative alignment and component positioning but did not validate full planned-versus-achieved implementation of the preoperative software plan. Intraoperative accuracy, soft-tissue balance, intramedullary canal violation, intraoperative blood loss, implant micromotion, and long-term fixation were not directly measured [24, 25]. Fifth, the AI-assisted component was a planning-support workflow rather than an autonomous system. The software-generated virtual postoperative joint motion state was an intra-planning simulation, not true postoperative clinical ROM data, and the simulated motion state was not validated against actual postoperative clinical ROM. Postoperative flexion angle and total arc of motion were not systematically collected; therefore, complete clinical ROM comparison could not be analyzed. Sixth, gait findings were based on a very small sample and should be regarded as exploratory and descriptive rather than definitive evidence of clinically meaningful gait benefit. GPS lacks universally accepted clinically important thresholds after TKA, and the GPS reference dataset was derived from healthy young adults aged 23–25 years rather than from an age-matched older adult population. Seventh, HALP is not a validated TKA-specific endpoint and should be interpreted only as an exploratory laboratory index. The CT-based workflow also requires radiation exposure, software planning, data transfer, guide printing, post-processing, sterilization, coordination resources, and additional lead time, and no formal cost-effectiveness or step-level workflow-time analysis was performed. Finally, the follow-up period was short, and KOOS data were not prospectively collected. Larger randomized or well-matched prospective studies with predefined primary endpoints, stronger confounder control, predefined radiographic alignment and component-positioning outcomes, longer follow-up, and health-economic evaluation are needed.

### Balanced clinical adoption

A balanced clinical approach remains necessary when considering AI-assisted planning and patient-specific cutting guide workflows. These technologies should be viewed as adjuncts to, not replacements for, surgical judgment. Surgeons should weigh the additional imaging, planning, cost, resource requirements, and waiting time against the potential benefits in carefully selected patients.

## Conclusion

In this prospective exploratory cohort of 22 patients undergoing primary unilateral TKA, AI-assisted three-dimensional preoperative planning with patient-specific cutting guides was associated with observed short-term differences in selected functional, perioperative, radiographic component-positioning, and gait outcomes compared with conventional planning and instrumentation. Quantitative radiographic analysis showed smaller coronal tibial component error in the AI-assisted planning/PSI group, while overall postoperative HKA deviation and HKA outlier rates were comparable between groups. The main practical role of the workflow was to allow preoperative software simulation, repeated virtual osteotomy review, implant-size and implant-position review, and virtual motion-state visualization before guide fabrication. Because the study was small, single-center, non-randomized, and involved multiple nominal comparisons that should be interpreted as exploratory and descriptive rather than confirmatory, it does not establish causal superiority, definitive clinical benefit, or validated planned-versus-achieved accuracy. The workflow appears feasible within the present institutional setting, but larger studies with stronger confounder control, predefined primary endpoints, longer follow-up, and cost-effectiveness analysis are required.

## Supplementary Information

Below is the link to the electronic supplementary material.

Supplementary file1 (PDF 621 KB)

## Data Availability

The datasets generated and/or analyzed during the current study are available from the corresponding author on reasonable request.
