# Supplementary material for: Artificial intelligence-assisted three-dimensional preoperative planning with patient-specific cutting guides versus conventional planning and instrumentation in total knee arthroplasty: a prospective exploratory cohort study
Source: J Orthop Surg Res. 2026 Aug 14;21:635. doi: 10.1186/s13018-026-07132-4 (PMC13753087; doi:10.1186/s13018-026-07132-4)
Supplement: Supplementary file 1 [file 13018_2026_7132_MOESM1_ESM.pdf]

Supplementary Table 1. STROBE checklist for cohort studies

Note. Line numbers refer to the manuscript R5. N/A = not applicable.

Section	Item	STROBE recommendation	Reported location in manuscript R5	Reporting details
Title and abstract	1(a)	Indicate the study design using a commonly used term in the title or abstract.	Title, lines 1-3; Abstract, lines 37-42.	Reported as a prospective exploratory cohort study.
Title and abstract	1(b)	Provide an informative and balanced summary of what was done and what was found.	Abstract, lines 29-54.	Reported study rationale, design, sample size, outcomes, key results, and cautious conclusions.
Introduction	2	Explain the scientific background and rationale for the investigation.	Introduction, lines 58-90.	Reported clinical background, digital planning rationale, gait-analysis rationale, and HALP rationale.
Introduction	3	State specific objectives, including any prespecified hypotheses.	Introduction, lines 91-96.	Reported the objective as describing observed between-group differences and workflow feasibility, not causal superiority.
Methods	4	Present key elements of study design early in the paper.	Abstract, lines 37-42; Methods, lines 99-105.	Reported single-center prospective exploratory cohort design.
Methods	5	Describe setting, locations, relevant dates, recruitment, exposure, follow-up, and data collection periods.	Methods, lines 99–105; allocation and exposure, lines 112–134; AI/PSI workflow, lines 135–200; data collection and outcome assessment, lines 261–296; clinical follow-up timing, lines 270–271	Reported tertiary orthopedic center, March-May 2025 enrollment, non-randomized allocation pathway, AI/PSI exposure, and preoperative plus 2- and 6-month postoperative assessments.
Methods	6(a)	Give eligibility criteria, and the sources and methods of selecting participants. Describe methods of follow-up.	Eligibility criteria, lines 106–111; participant selection and allocation, lines 112–134; clinical follow-up timing, lines 270–271.	Reported inclusion/exclusion criteria, surgeon-guided selection, AI-assisted pathway flow, Conventional comparison group, and follow-up assessments.
Methods	6(b)	For matched studies, give matching criteria and number of exposed and unexposed participants.	Not applicable.	No matching design was used; the study was a non-randomized exploratory cohort.
Methods	7	Clearly define outcomes, exposures, predictors, potential confounders, and effect modifiers. Give diagnostic criteria where applicable.	Allocation/exposure, lines 112–134; AI/PSI workflow, lines 135–200; surgical procedures, lines 220–260; outcomes, lines 261–296; gait variables, lines 310–344; confounding and selection bias, lines 666–676.	Defined the exposure as AI-assisted planning/PSI versus conventional instrumentation; defined clinical, perioperative, radiographic, and gait outcomes; acknowledged selection-related confounding.
Methods	8	For each variable of interest, give data sources and details of assessment or measurement methods.	CT/planning workflow, lines 136–200; clinical, perioperative, ROM, and radiographic outcome definitions, lines 261–296; blinded assessment, lines 305–309; gait analysis, lines 310–344; statistical methods, lines 345–372.	Reported CT acquisition, software-assisted planning, clinical scores, pain, drainage, radiographic measurements, gait processing, and analytic methods.
Methods	9	Describe efforts to address potential sources of bias.	Allocation and selection, lines 112-119; blinded assessment and standardized care, lines 305-309; statistical approach, lines 345-372; limitations, lines 666-696.	Reported non-randomized selection, blinded outcome assessment, standardized care pathways, nominal/exploratory interpretation, and limitations related to selection bias and confounding.
Methods	10	Explain how the study size was arrived at.	Abstract sample size, lines 37-39; participant flow/results, lines 375-380; statistical methods, lines 370-372.	Reported the analyzed sample size and stated that no formal sample size calculation was performed because of the exploratory design.
Methods	11	Explain how quantitative variables were handled in analyses. If applicable, describe groupings.	Outcome definitions, lines 261-296; statistical methods, lines 345-364; radiographic variables, lines 288-296.	Reported use of mean +/- SD, medians/interquartile ranges, HKA deviations, component errors, risk differences, and confidence intervals/effect estimates where estimable.
Methods	12(a)	Describe all statistical methods, including those used to control for confounding.	Statistical methods, lines 345-372; HKA sensitivity-analysis method, lines 357-364.	Reported normality testing, t tests, Mann-Whitney U tests, chi-square/Fisher tests, repeated-measures analyses, effect sizes, radiographic exact tests, and exploratory baseline-adjusted HKA sensitivity analysis. No multivariable adjustment, propensity-score matching, or weighting was performed for the main clinical outcomes because of the limited sample size.
Methods	12(b)	Describe methods used to examine subgroups and interactions.	Repeated-measures method, lines 365-366; repeated-measures results, lines 425-427; subgroup limitation, lines 672-673.	Reported group-by-time interaction testing; subgroup analyses were limited by small sample size.
Methods	12(c)	Explain how missing data were addressed.	Complete-data criterion, lines 106-108; participant flow, lines 129-134 and 375-380; ROM data availability, lines 280-287; KOOS limitation, lines 692-693.	Reported complete-data eligibility, loss to follow-up/exclusion, unavailable full clinical ROM data, and absence of prospectively collected KOOS data.
Methods	12(d)	If applicable, explain how loss to follow-up was addressed.	Participant flow, lines 129-134; results, lines 375-380.	Reported one patient excluded because of loss to follow-up and final analyzed numbers in each group.

Section	Item	STROBE recommendation	Reported location in manuscript R5	Reporting details
Methods	12(e)	Describe any sensitivity analyses.	Statistical methods, lines 357–364; results, lines 498–503.	Reported exploratory sensitivity analysis of postoperative HKA deviation adjusted for baseline absolute preoperative HKA deviation.
Results	13(a)	Report numbers of individuals at each stage of the study.	Participant flow, lines 129-134; results, lines 375-380.	Reported 15 patients counselled for AI-assisted planning/PSI, exclusions/declines/loss to follow-up, and final 11 per group.
Results	13(b)	Give reasons for non-participation at each stage.	Participant flow, lines 129-134 and 375-380.	Reported perioperative complications, non-selection of the workflow, and loss to follow-up.
Results	13(c)	Consider use of a flow diagram.	Participant flow described in text, lines 129-134 and 375-380.	No separate flow diagram was included; participant flow was reported in the text.
Results	14(a)	Give characteristics of study participants and information on exposures and potential confounders.	Allocation description, lines 112-119; baseline description and Table 1 citation, lines 381-393.	Reported demographic/clinical baseline characteristics and noted borderline baseline KSS difference and lack of proof of baseline equivalence.
Results	14(b)	Indicate the number of participants with missing data for each variable of interest.	Complete-data criterion, lines 106-108; participant flow, lines 129-134 and 375-380; ROM data availability, lines 280-287.	Reported that complete perioperative data and follow-up were required; one patient lost to follow-up was excluded; complete clinical ROM was not systematically available.
Results	14(c)	Summarize follow-up time.	Abstract, lines 39-40; clinical follow-up, lines 270-271.	Reported preoperative and 2- and 6-month postoperative assessment time points.
Results	15	Report numbers of outcome events or summary measures over time.	Clinical/pain outcomes, lines 404-439; inflammatory/perioperative outcomes, lines 441-476; radiographic outcomes, lines 477-518; gait outcomes, lines 520-563.	Reported outcome summaries across clinical, perioperative, radiographic, and gait domains.
Results	16(a)	Give unadjusted estimates and, if applicable, confounder-adjusted estimates with precision.	Main results, lines 404-543; HKA sensitivity analysis, lines 498-503.	Reported median/mean differences and approximate or exact 95% confidence intervals; reported adjusted HKA sensitivity coefficient.
Results	16(b)	Report category boundaries when continuous variables were categorized.	Severe pain definition, lines 278-279; radiographic outlier definition, lines 288-292 and 489-497.	Reported NRS >=7 for severe pain and >3 degrees for radiographic outliers.
Results	16(c)	If relevant, translate relative risks into absolute risks over a meaningful time period.	Radiographic outlier reporting, lines 489-490; Table 6 note, lines 513-518.	Reported HKA outlier rates and risk differences rather than relative measures alone.
Results	17	Report other analyses done, such as subgroup analyses, interactions, and sensitivity analyses.	Repeated-measures results, lines 425-427; sensitivity analysis, lines 498-503.	Reported group-by-time interactions for WOMAC, KSS knee score, and KSS function score; reported HKA sensitivity analysis.
Discussion	18	Summarize key results with reference to study objectives.	Discussion, lines 565-579; conclusion, lines 702-715.	Summarized feasibility and selected observed short-term differences while avoiding causal claims.
Discussion	19	Discuss limitations, considering potential sources of bias or imprecision.	Limitations, lines 666-696.	Reported non-randomization, selection bias, small sample, multiple comparisons, unvalidated planned-versus-achieved implementation, incomplete ROM, gait/GPS limitations, HALP uncertainty, CT/resource demands, no cost-effectiveness analysis, short follow-up, and no KOOS.
Discussion	20	Give a cautious overall interpretation of results considering objectives, limitations, multiplicity, and related evidence.	Discussion, lines 565-665; conclusion, lines 702-715.	Interpreted findings as exploratory, non-causal, and hypothesis-generating; emphasized comparable HKA alignment and need for validation.
Discussion	21	Discuss generalizability of study results.	Clinical applicability, lines 643-665; balanced adoption, lines 697-701; limitations/future directions, lines 666-696.	Discussed dependence on CT, software, engineering support, 3D printing, sterilization, lead time, and resource-limited settings.
Other information	22	Give the source of funding and the role of funders.	Funding statement, lines 729-736.	Reported funding sources and stated that funders had no role in study design, data collection, analysis, interpretation, or manuscript writing.
